# Governing and Measuring Health Security: The Global Push for Pandemic Preparedness Indicators

**DOI:** 10.1111/1758-5899.13090

**Published:** 2022-04-26

**Authors:** Alexander E. Kentikelenis, Leonard Seabrooke

**Affiliations:** ^1^ Bocconi University Milan Italy; ^2^ University of Cambridge Cambridge UK; ^3^ Copenhagen Business School Frederiksberg Denmark; ^4^ Norwegian Institute of International Affairs Oslo Norway

## Abstract

Providing collective solutions to global pandemics requires the coordination of information that is accurate and accountable. In recent years there has been a global push for reliable pandemic preparedness indicators. This push has come from U.S. foreign policy, the World Health Organization (WHO), NGOs, and private foundations. These actors want control over how data for preparedness indicators is collected, analysed, and promoted. Governments want to influence how they are assessed, using poor performance to attract attention and good performance to deflect blame. In this article we discuss how the push for pandemic preparedness indicators comes from the dual aims of repelling national risk, the spread of disease, while reducing global harm through stronger transnational governance arrangements. We delve into the development of indicators from the WHO and the privately‐run Global Health Security Index, and examine how their claims to authority measure‐up against standards of transparency, veracity, and accountability. We stress the importance of understanding how these indicators are composed. This is vital given the current drive to include social and governance metrics in revised efforts at data collection, as well as efforts to include pandemic preparedness indicators in how intergovernmental organizations, NGOs, donors, and funders devise health and development policies.

## 1 THE PROBLEM OF MEASUREMENT IN PANDEMIC PREPAREDNESS

Without measurement, there can be no improvement. This was the famous dictum by 19th century scientist Lord Kelvin, and it has almost become a truism in contemporary policy debates, where the problem is no longer lack of measurement but the presence of multiple, often overlapping measurements of policy environments and infrastructures. In principle, such measurements can—and often do—contribute to more effective policy design. Yet, not all aspects of policy infrastructures can be captured in indicators, and sometimes—in the face of uncertainty and complex causality—indicators may be neglecting what is important for what is easily measurable.

The current experience with the Covid‐19 pandemic provides a case in point. For more than a decade, the global policy community has sought to measure how well‐prepared different countries are for a pandemic through indicators. Intergovernmental organizations (IGOs), NGOs, and large donors have pushed the demand for such indicators as means to provide simplified quantitative representations of complex scenarios (Rottenburg & Merry, 2015:3). However, when a pandemic actually emerged, available indicators generally failed to map onto how countries fared. Supposedly highly prepared countries in the Global North—like the U.S. or the U.K.—witnessed high degrees of infection and death in the early stages of the pandemic, while countries with comparably lower resources—like Vietnam or Thailand—managed to initially limit the spread of the novel pathogen. This prompted influential observers like Branko Milanovic (2021) to quip that indicators that sought to inform the world on pandemic preparedness ‘either entirely failed, or can be shown to have been useless.’

While we do not share this limiting view on the role and functions of indicators, it does beg important questions: how can pandemic preparedness be measured, and how can measurements be improved to capture the underlying policy realities? These questions matter for three reasons. First, pandemic preparedness indicators feed directly into the political salience and public valence of health emergency responses. For example, in the early months of Covid‐19, President Trump boasted that the U.S. was ranked first in the Global Health Security Index (discussed below) and provided it as evidence of why no alarm was necessary. As this anecdote suggests, preparedness indicators can be used as political tools and can be considered as the ‘enactment of a form of global biopolitics’ (Lakoff, 2022:30). Second, pandemic preparedness indicators are central in discussions among transnational policy and scientific communities on how to improve responses to health emergencies and what issues need prioritization. What is included in these indicators reflects a combination of political and scientific priorities (Fukuda‐Parr & McNeill, 2019). Finally, pandemic preparedness indicators are rapidly being institutionalized: IGOs, non‐governmental organizations, and private actors are urging development‐focused organizations to use preparedness indicators in their funding decisions. This means that these metrics have an increasing—even alarming—influence on policy outputs.

In this article, we examine the promises and pitfalls of pandemic preparedness indicators, with an emphasis on the three main publicly available indicators with cross‐national coverage: the State Parties Annual Reporting (SPAR) that relies on government‐reported data, the WHO‐administered Joint External Evaluations (JEE), and the privately‐run Global Health Security Index (GHSI). Our findings draw both on our analysis of the content and methodologies of these indicators, as well as a review of academic and policy literature. We complement these data sources with nine interviews with policymakers and experts centrally involved in these global measurement processes.

Overall, our analysis draws attention to three key policy issues pertaining to the measurement of pandemic preparedness. First, all three indicators reflect distinct ways of organizing knowledge, and—correspondingly—they have blind spots vis‐à‐vis measurement. This is unsurprising, but it introduces important biases insofar as the indicators are used to compare countries with widely differing policy environments and political cultures. In other words, the indicators are ultimately acts of ‘commensuration’ (Espeland & Stevens, 1998), whereby different data sources are fed into a single indicator that claims internal validity notwithstanding doubts about underlying comparisons.

Second, the emergence of pandemic preparedness indicators can be traced back to a mix of foreign policy objectives—primarily by states in the Global North—on health security (i.e., the desire to repel national risk from the spread of communicable disease) and global health policy objectives (i.e., to reduce global harm). The dual, occasionally competing objectives lead to important challenges in the construction of indicators, including how global health security is treated as a ‘measurable condition’ (Lakoff, 2022:27). These challenges are not only scientific but also political, as the construction of indicators shapes policy decisions—like the allocation of global health funding—and therefore has politico‐economic distributive consequences (cf. Fukuda‐Parr et al., 2014).

Finally, pandemic preparedness indicators are gaining traction in a transnational policy environment in which different intergovernmental and non‐governmental organizations are looking for anchors to coordinate their actions. In some policy discussions between organizations like the WHO, the World Bank, and the Bill and Melinda Gates Foundations, these indicators provide a common talking point that creates a demand for more data that can be operationalized. As such, current pandemic preparedness indicators follow trends associated with other global health issues—such as malaria and HIV—in being associated with organizational and national administrative efficiencies (Gerrets, 2015:154; Park, 2015:211). Revisions to the GHSI to include social and governance metrics present a most recent example. These include newly developed indicators on ‘risk environment’ that measure the quality of a bureaucracy and ‘norms’ that capture contributions to the WHO. Such indicators aim to judge a country's commitment to the current international order, as well as assessing their bureaucratic capacity to deliver emergency health services—these are politically loaded questions. In short, pandemic preparedness indicators need investigation because of how they are composed, what agendas they carry, and what momentum they bring to policy direction.

## 2 THE GENESIS AND EVOLUTION OF PANDEMIC PREPAREDNESS INDICATORS

International concern with the spread of infectious pathogens is not new. However, the policy lens employed in approaching infectious disease issues changed considerably over the decades: from being seen as a policy problem that can be ameliorated through extensive investments in primary care (as was the case with the rights‐based and development‐oriented Health for All approach in the late 1970s and 1980s), to being treated primarily as a security concern that should be pre‐empted through clear policy action by states (Chorev, 2012; Weber, 2020). Indeed, that was the raison d'être behind the International Health Regulations (IHR), a piece of binding international law in global health that has its roots in the 1952 International Sanitary Regulations before being renamed in 1969 (Fidler, 2015).

The way that IHR were put into practice changed over the years but remained primarily focused on a short list of infectious diseases. However, the emergence of pathogens that could much more rapidly spread across borders—partly a function of the much greater international movement of individuals and goods associated with globalization—sparked policy debates around the turn of the millennium on how to make IHR fit for purpose. Through this revision process that was agreed in 2005 and came into effect in 2007, the international community—acting through the WHO's World Health Assembly—sought to ramp up transnational coordination and surveillance of infectious disease through a security lens that reflected a ‘new political direction grounded in the core self‐interests of states’ (Fidler, 2015:183). This meant that much evidence needed to be gathered and systematized on disparate health policy and disease surveillance arrangements in all countries.

The securitization of global health created a functional need for cross‐national knowledge generation and subsequent policy action: the trope—commonly repeated during the Covid‐19 pandemic (UNSG, 2020)—that the world is only as safe as the weakest link meant that the international community should be able to identify weak links among countries and nudge or compel them into rectifying these gaps. Inadequate capabilities or insufficient action by one country to control the rise and spread of a new infectious disease could spell disaster for the world. Even so, IHR enforcement capacity remained constrained, with the key mechanism to induce compliance being ‘public shaming techniques that highlight damaged international reputations, increased national mortality, economic disruptions, and public outrage’ (Tonti, 2020).

Central in these efforts to upgrade the health emergency preparedness infrastructures of countries was the building up of ‘core capacities’ to prevent and detect infectious disease and provide a public health response to control it and halt its spread across borders. The IHR spell out eight such capacities, relating to laboratories, human resources, surveillance, preparedness, response to a health emergency, risk communication, coordination, and the adequacy of national legislation and policies. These are complemented by five additional capacities related to specific hazards (like food safety) and to controlling disease at points of entry. Compliance with these capacities would be based on countries' self‐assessments using the IHR Monitoring Questionnaire, launched by the WHO in 2010 and relying on yes/no answers.

This system became the established monitoring framework for IHR compliance (although revamped after 2018, as discussed below). The country‐submitted indicators were treated as official data by the WHO, and—despite occasional doubts over their veracity—were regularly used by the organization for its reports to the World Health Assembly (Kentikelenis & Seabrooke, 2021). However, the WHO lacks a legal mandate to verify the scores provided by countries, thereby casting doubts on the accuracy of the data and ensuing analysis. This limitation prompted influential observers to question the foundations of the entire enterprise: self‐assessments were seen as ‘inherently self‐interested and unreliable’ (Gostin & Katz, 2016) and many governments did not have the capacity to collect the required evidence in systematic ways.

These limitations became only more apparent in the Ebola crisis, where major global policy actors accepted the limits of IHR self‐reporting. As an alternative, many countries banded together to form the Global Health Security Agenda (GHSA). This was an initiative of the Obama administration in the U.S. in the immediate aftermath of Ebola, and relied on an initial partnership with another 43 countries to develop ‘an implementation vehicle to assist countries in achieving core capacities agreed to in the IHR’ on a voluntary basis (Kerr, n.d.). The lynchpin of this effort was the development of an evaluation template that could be uniformly applied to different countries by external experts, thereby overcoming the validity and reliability concerns that marred IHR self‐reporting. Under the initial Finnish presidency of the GHSA and the motto ‘What gets measured, gets done’ (Sillanaukee, 2015), the GHSA developed proof‐of‐concept assessments of five initial countries in 2015, with plans for a further rollout in subsequent months.

The GHSA expert‐based approach was a major development in attempts to measure pandemic preparedness, yet one that marginalized the WHO even while proclaiming commitment to its IHRs (Gostin & Katz, 2016). While the GHSA activities claimed to be ‘in support of international standards set by the World Health Organization, the Food and Agriculture Organization of the United Nations, and the World Organization for Animal Health’ (White House, 2014), these organizations only had an advisory status within the GHSA. This prompted serious concerns within the WHO about a possible loss of competencies related to its mandate, and the organization successfully lobbied for folding these expert advisory activities into its remit, although it had to rely on funding from the Bill and Melinda Gates Foundation to cover the administrative costs (Interview 10/6/21). This was the birth of the Joint External Evaluations (JEEs): a system of country assessments based on a universally applicable template that is filled in by experts who are not nationals of the evaluated country and are drawn from a roster held by the WHO.

These WHO‐supported evaluations started being developed in 2016, and by 2021 more than 100 countries—mostly low‐ and middle‐income ones—had completed one. The process for developing the reports generally proceeds in two steps. First, there is a set of internal consultations within countries that commonly involve the participation of multiple ministries or agencies. After this phase is complete and the material required for the evaluation has been compiled, the JEE team visits the country for one week during which they review this material and hold meetings with relevant actors. Subsequently, the team drafts the report and assigns scores, which are transmitted to the government for input, although the latter cannot alter the content of the evaluation. According to participants in these processes, a key merit of the JEE is in bringing key policy actors together and facilitating hands‐on policy transfers from the international experts to the national officials (Interviews 6/5/2021, 2/6/2021). Tellingly, even though the JEE team assigns scores across a host of capacities, these scores are not readily available in the form of an accessible cross‐national dataset, but only referenced in the main text of the country report. In other words, unlike SPAR and GHSI, this type of expert evaluation does not easily lend itself to developing global rankings, instead emphasizing the qualitative nature of the analysis and the concrete policy recommendations.

While the JEE analyses are generally acknowledged as comprehensive, they are still seen by many users and stakeholders as reliant on expert judgement, being highly costly in terms of organization and coordination (but not financially, as external experts are not remunerated), and having limited use for cross‐country comparisons (Interviews 4/6/2021, 10/6/21). Indeed, for the latter purposes, the WHO still officially relies on self‐reporting by countries, which was revamped in 2018: following criticisms of the binary approach to capturing a complex underlying policy reality, the IHR Monitoring Questionnaire was replaced by the more comprehensive Electronic State Parties Self‐Assessment Annual Reporting Tool (e‐SPAR). This presents countries with a range of questions on IHR capacities, and they can score themselves along a continuum ranging from having no policies in place to implement IHR guidance to having such policies and strategies at multiple levels of policymaking and subject to regular updates.

Even though e‐SPAR reflects an analytical improvement from the previous modus operandi, it still faces challenges over the veracity of self‐reported information. To address these concerns, as well as the limitations of the JEE, a collaboration between the Johns Hopkins School of Public Health, security think‐tank Nuclear Threat Initiative, and private forecaster Economist Intelligence Unit created the GHSI, launched in 2019. Instrumental in the creation of the Index was Elizabeth Cameron, the former senior director for global health security and biodefense in the National Security Council under the Obama administration, who was also behind the launch of the Global Health Security Agenda, discussed above. This type of policy knowledge was joined by the academic expertise and data collection capacities of Index partners to develop the first comprehensive ranking system on pandemic preparedness.

Unlike the e‐SPAR's self‐reported scores by governments and the JEE's expert judgements, the GHSI avows such objective sources in favour of analyses of regulations and policies as they exist in the public domain. These are collected by country teams of the Economist Intelligence Unit, and subsequently submitted to the U.S.‐based research team who assess the quality of the information and assign scores, based on a scoring manual developed by the lead researchers in collaboration with a high‐profile international panel of experts.

As the initial GHSI scores were published just a few months prior to the emergence of Covid‐19, the pandemic offered scholars and practitioners a unique chance to examine the validity of the indicator. Could GHSI scores predict the types of responses that countries actually developed or public health outcomes? Early analyses were sceptical (Abbey et al., 2020; Milanovic, 2021), and close observers criticized the types of expertise that were prioritized and reflected in the GHSI (Dalglish, 2020). A further criticism has been that the GHSI relies on largely anticipatory categories based on a poorly theorized concept of global health security that is premised on a fear of new infectious diseases rather than strengthening capabilities to mitigate known ones (Mahajan, 2021).

The GHSI team themselves accepted limitations of the initial indicators (Ravi et al., 2020), and updated their methodology for the second iteration of the Index—published in end‐2021—to include more relevant variables, including on issues of political risk, social trust, and quality of information (GHSI, 2021b). This expansion of the remit of the indicator has emboldened the GHSI team to recommend the use of the Index to the WHO, the World Bank, and other global funders to ‘identify countries that may benefit most from additional support to improve their readiness for future disease emergencies, prioritizing assistance to countries with higher political and socioeconomic risk factors’ (GHSI, 2021a:60).

## 3 CHALLENGES IN MEASURING PREPAREDNESS

All indicators require the support of an authority willing to defend them and help them gain traction as policy tools. In our context, authority emanates primarily from two sources: formal authority from a mandated organization, such as an IGO supported by member states; and expert authority where those producing the indicator are viewed as credible in their capacity to judge effectively (cf. Littoz Monnet, 2022). Both forms of authority can lead the intended audience to defer to the judgement of the party producing the indicator and ‘obey’ the commands put forth, as in the classic Weberian definition (Weber, 1978:53). These forms of authority require legitimation in that there is an expressed belief from the indicator's audience that it is backed by political power or scientific prowess (Fukuda‐Parr & McNeill, 2019; Kentikelenis & Seabrooke, 2017). Without authority, no action is likely to result from any preparedness indicator. Commonly, such authority rests on claims of *transparency*, *veracity*, and *accountability* in relation to how an indicator is constructed. Lacking these trains undermines claims to authority. As such, authority sits ‘above’ standards of transparency, veracity, and accountability.

The three indicators noted above—e‐SPAR, JEE and GHSI—can be assessed in terms of how they measure up to standards of transparency, veracity, accountability. It is unlikely that a single indicator will meet a high standard across all three elements. The political and scientific compromises made to compose the indicator will reflect different combinations of these three elements, which we can consider conceptually and examine empirically.

To begin, there is the issue of how identifying what data was collected, and who collected it. This issue of *transparency*, an important global norm of the past twenty years among intergovernmental and non‐governmental organizations (Kim & Sharman, 2014), is fundamental for the external recognition and legitimacy of pandemic preparedness indicators. If the process of collecting the data is subject to political inference or obvious bias then the legitimacy of the indicator can be attacked, casting it as narrowly in the category of repelling national harm and not of service to reducing global harm.

A crucial test for pandemic preparedness indicators is the *veracity* of the data used to construct them: how truthful, accurate, and reliable is it? Data veracity can be impaired by poor coding processes where values are mis‐assigned or misinterpreted, or through the introduction of biases to make data comparable when comparisons are inappropriate (Bragazzi et al., 2020). In the case of pandemic preparedness indicators, achieving veracity is difficult because preparedness is itself not clearly defined but an ‘imaginative enactment’ based on a mix of policy objectives (Lakoff, 2022). Particularly important, then, is how questions asked then arrive at standardized answers, and whether adding more data points strengthens or weakens truthfulness. Problems in maintaining a high standard of veracity are particularly acute when those handling the data have shallow knowledge of what the data *means*, or where there is political inference for the data to reflect a predetermined outcome (Jerven, 2013). Problems with data veracity can be compounded when data inputs are cobbled together from different sources, and where the aims of original data collection differ from their aims of the indicator they then contribute towards. The emphasis placed on what one aspect of data contributes compared to another requires those composing the indicator to factor in what is truthful and accurate, which can be compromised when knowledge about the different data sources is weak. Problems with overseeing different kinds of data introduce a ‘politics of variability’ in how data sources are treated and mediated with stakeholders, a process seen in other aspects of global public health, such as access to medicines indices (Mehrpouya & Samiolo, 2016; Nilsson, 2017).

While transparency and veracity refer to the processes of data collection and accuracy, there are also concerns with whether or not the party creating the indicator is *accountable*. Concerns over accountability include whether or not the indicator created by the party can be challenged and potentially revised and whether stakeholders have any voice: who accounts for the outcomes produced by indices, benchmarks, and other forms of ranking? In the case of the GHSI, accountability issues are especially acute given the links between rankings from the Index and intended funding outcomes from IGOs like the World Bank and from philanthropic organizations, like the Bill and Melinda Gates Foundation. Given that governments have little input into the data collected by the GHSI, they may complain that their policies have been misunderstood or misrepresented and that those composing the Index are not accountable, especially given the partnership is a composite of a security NGO, an economic think‐tank, and a university. The GHSI's current line of reasoning is that the Index can motivate ‘mutual accountability, encourage transparency, and urge progress toward a safer world’ by revealing to IGOs, donors and the private sector where countries need help (GHSI, 2021a:10), while its own accountability draws heavily on the prestige of its international panel of experts that support and oversee the coding practices.

**FIGURE 1:**
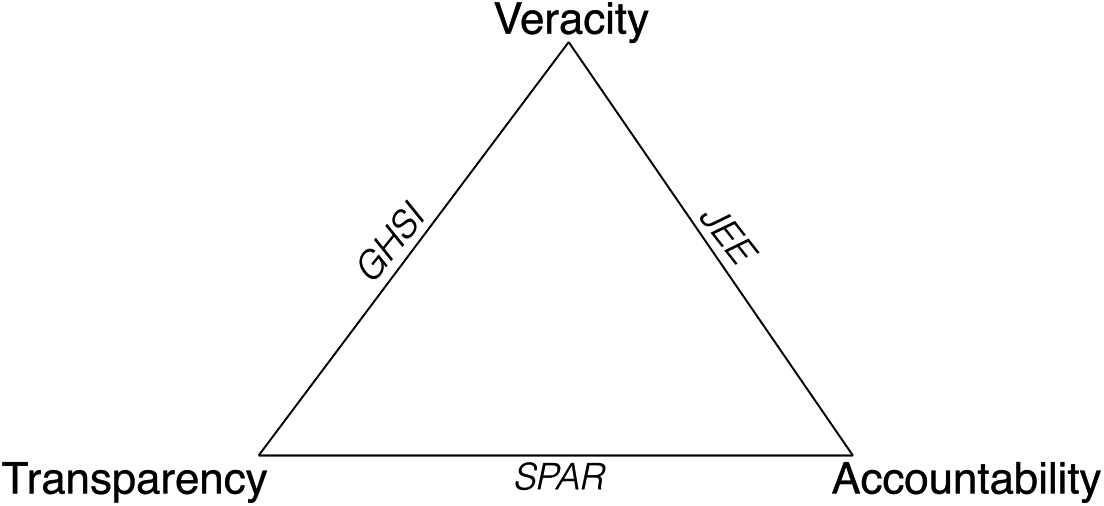
The pandemic preparedness indicator trilemma

This discussion generates a question as to whether there is a trade‐off among transparency, veracity, and accountability in supporting the authority of indicators. Figure 1, below, presents transparency, veracity, and accountability as choices in a trilemma and locates the indicators discussed above. In all cases there is a selection of two aspects to the exclusion of a third. The e‐SPAR indicator is transparent in the process of collecting metrics and accountable to the national governments (as they generate and report the data), but there are serious doubts about the veracity of the data underpinning the indicators. The JEE does well in combining veracity, given the high‐level of expertise, and accountability, given their WHO mandate and scientific recognition, but cannot make strong claims on transparency since much of the purpose of missions is to create confidential dialogue. The GHSI's process is transparent and veracity is ensured through the collection of all available public data that is then referenced in the justifications for each score. But the Index is criticized for not being accountable in not sufficiently allowing those being ranked to object. As it stands, there are currently no pandemic preparedness indicators that have strong overlapping claims to transparency, veracity, and accountability.

## 4 RETHINKING MEASUREMENT

The development of pandemic preparedness indicators has sought to combine two objectives. The first is to reduce *national risk*, which is easily identified in the Obama administration's embracing a ‘health security’ framing of global health in response to the Ebola crisis (Fidler, 2015). Since that response, the U.S. has exercised political leadership in integrating health security into IGO, NGO, and private foundation activities on global public health. This is a marriage with the second objective—to reduce *global harm* by developing collective capacities to boost emergency health preparedness. In principle, preempting national risk and reducing global harm are not competing policy objectives. However, depending on a government's capacities and dependencies, policies undoubtedly favour one over the other. Choosing the mix of these objectives involves a series of trade‐offs.

The first trade‐off is the selection of policy bias towards preempting national risk or reducing global harm. While some countries—especially high‐income ones—can combine these objectives, most countries need to decide how to allocate scarce resources in favour of one over the other. Judging from the Covid‐19 response, there is no simple measure by which we can determine the balance between the two objectives. Even in seemingly similar Scandinavian countries, the choice to favour national or global objectives has diverged according to politico‐administrative interests (Laage‐Thomsen & Frandsen, 2022).

The second trade‐off relates to political choices made in the compilation of indicators. It is difficult to collect data and compose an indicator that satisfies high standards for transparency, veracity, and accountability. Those propagating preparedness indicators are forced to place emphasis on two of these three, and this is a process that is both political and scientific. These choices have implications for the political reception of indicators and for the integrity of the scientific community that supports their interpretation.

The final trade‐off is between visions of global health and whether they should favour a security frame (‘protection from’) or a human rights frame (‘protection for’). Zooming out from the current pandemic, we encounter this trade‐off in earlier debates around the WHO's Health for All strategy centered on health as a universal human right and how the IHR were developed with a strong conception of national interest in mind (Chorev, 2012; Fidler, 2015). The development of pandemic preparedness indicators makes this trade‐off starker in allowing policy audiences to easily compare and judge national performance—who is worthy and who is failing?—through either the lens of security or universal care.

Where does all this leave attempts at measuring pandemic preparedness? Indicators have a great role to play as informative aids for countries themselves to track progress and identify problems to be remedied. However, when they are uncritically used by third actors—including donor agencies or international organizations—to decide on where to allocate resources or make other consequential decisions, they can ultimately fail to deliver on their promise and might lead to misdiagnoses of needs and priorities.

## Data Availability

Data sharing is not applicable to this article as no new quantitative data were created or analyzed in this study.
